# Development and Validation of a Perception, Attitude, and Practice of Physical Activity to Support Personalized Physical Activity Promotion Among U.S. Older Adults

**DOI:** 10.3390/sports14020081

**Published:** 2026-02-13

**Authors:** Oluwaseun Adeyemi, Dowin Boatright, Joshua Chodosh

**Affiliations:** 1 Department of Emergency Medicine, New York University Grossman School of Medicine, New York, NY 10016, USA; 2 Department of Population Health, New York University Grossman School of Medicine, New York, NY 10016, USA; 3 Department of Medicine, New York University Grossman School of Medicine, New York, NY 10016, USA; 4 Veterans Affairs New York Harbor Healthcare System, New York, NY 10010, USA

**Keywords:** perception, attitude, practice, physical activity, older adults, instrument validation

## Abstract

Background: This cross-sectional study aimed to develop and validate measures of perceptions, attitudes, and practices to support physical activity among older adults. Method: We enrolled online 310 community-dwelling U.S. older adults and 11 content experts. Using the Knowledge, Attitudes, and Practices framework, we developed 14, seven, and nine items for the Perceived Physical Activity Benefits Scale (PBAS), Attitudes toward Physical Activity Scale (APAS), and Physical Activity Practice Scale (PAPS), respectively. We generated derivation and replication samples using a 30:70 simple random split. Content validity and item analyses were performed on the full sample, followed by exploratory factor analysis (EFA) and confirmatory factor analysis (CFA) for derivation and replication samples, respectively. Results: Item-level content validity indices for the PBAS, APAS, and PAPS were 0.96, 0.94, and 0.95, respectively. Also, the internal consistencies for the PBAS, APAS, and PAPS were 0.92, 0.77, and 0.91, respectively. Our EFA identified two subscale constructs for each measure, with good subscale reliability. CFA fit index ranges for the PBAS, APAS, and PAPS were 0.90–0.94, 0.97–0.99, and 0.95–0.97. Conclusions: The PBAS, APAS, and PAPS are reliable and valid instruments for assessing perceptions, attitudes, and practices related to physical activity among older adults.

## 1. Introduction

Physical activity is a cornerstone of healthy aging, yet inactivity remains highly prevalent among U.S. older adults [1,2,3]. Despite well-established evidence that regular physical activity reduces the risk of cardiovascular disease, frailty, falls, cognitive decline, and all-cause mortality among older adults [4,5,6,7,8], engagement in physical activity remains low [9,10]. Physical inactivity contributes to loss of independence, diminished quality of life, and increased healthcare costs [11,12,13]. Conversely, consistent engagement in moderate-intensity activities, such as walking, cycling, or resistance training, has been shown to improve balance, strength, mood, and cognitive function [1,14,15].

To improve physical activity among older adults, the World Health Organization and the U.S. Department of Health and Human Services recommend that adults aged 65 years and older engage in at least 150 min of moderate-intensity or 75 min of vigorous-intensity aerobic activity weekly, along with muscle-strengthening and balance exercises [16,17]. However, adherence to these recommendations remains poor. As of 2022, 14 percent of older adults met the physical activity guideline, with the proportion decreasing from 16 percent among those aged 65 to 74 years to six percent among those aged 85 years and older [18]. This reduced engagement in physical activity may reflect both physical limitations and psychosocial barriers, such as fear of falling, lack of motivation, and limited social support [19,20,21]. Previous studies have shown that older adults often have general awareness of physical activity’s health benefits but may underestimate the amount or intensity required to achieve them [22,23]. Misconceptions about safety, particularly regarding exercise-related injury or fatigue, further discourage participation [24]. These findings underscore the need for approaches that account for individual differences in perceptions, attitudes, and behavioral readiness when promoting physical activity among older adults.

To address individual-level differences in perception, attitudes, and practices towards physical activity among older adults, several validated instruments have been developed [25,26,27,28]. However, each instrument addresses only specific domains and subdomains of knowledge about the role, benefits, and recommendations for physical activity, attitudes toward engaging in physical activity or exercise, or physical activity practices. The Exercise Benefits and Barriers Scale focuses on perceived advantages and constraints related to exercise but does not assess attitudes or practice behaviors [28]. The Behavioral Regulation in Exercise Questionnaire and its modifications emphasize motivational regulation and motivation but do not assess knowledge or patterns of practical engagement [25]. Also, the International Physical Activity Questionnaire provides reliable estimates of physical activity levels but offers no insight into the underlying beliefs, attitudes, or knowledge that drive behavior [26]. A critical gap therefore exists: no validated instrument comprehensively assesses perception, attitudes, and practices within a unified framework, limiting integrated assessment and targeted intervention design for older adults.

To address this gap, this study aims to develop and validate measures of perceptions, attitudes, and practices to support personalized promotion of physical activity among older adults. Specifically, this study has three objectives: (1) develop items that measure perceptions, attitudes, and practices related to physical activity among U.S. older adults using the Knowledge, Attitudes, and Practices framework; (2) establish content validity through expert evaluation; and (3) examine the reliability and factor structure of each measure. We address the following research questions: (1) Do the instruments demonstrate adequate content and construct validity? (2) Do the instruments demonstrate adequate reliability? (3) What are the factor structures of each instrument? By assessing the reliability and validity of these measures, we seek to provide standardized instruments that capture individual-level variation in perceptions, attitudes, and practices related to physical activity among U.S. older adults.

## 2. Materials and Methods

### 2.1. Research Design

We conducted an online cross-sectional survey of community-dwelling older adults and content experts enrolled in the survey development and validation phase of the Activity Tracking, Care Partner Co-Participation, Text Reminders, Instructional Education, Virtual Physical Therapy, and Exercise (ACTIVE) study. The ACTIVE pilot study is a two-arm randomized intervention aimed at improving physical activity among older adults [29]. The study is registered on ClinicalTrials.gov (NCT07321587, Registration date: 23 December 2025) [30]. Prior to the start of the pilot study, we recruited participants for this survey development and validation through ResearchMatch, a nationwide online research registry that connects investigators with individuals interested in participating in research [31]. ResearchMatch has over 120,000 volunteers, including over 13,000 older adults [31]. Recruitment invitations were distributed via the platform’s email listserv, and individuals interested in participating provided their email addresses for screening, eligibility, and electronic consent before gaining access to the survey. Data were collected using REDCap (version 15.5.32), a secure, web-based platform for research data capture and management [32,33].

### 2.2. Participants

Eligible participants were adults aged 65 years or older who resided in the community, were able to complete an online questionnaire in English, and provided informed consent. Older adults residing in long-term care facilities and with a history of dementia were excluded. Content experts were similarly recruited from ResearchMatch and were required to have advanced degrees (Master’s, PhD, or MD) and at least three years of research experience in public health, health services, aging, physical activity, rehabilitation, or caregiving research. Consistent with established recommendations of 5 to 10 experts for content validation studies [34], we aimed to recruit a maximum of 15 experts to account for incomplete survey responses.

We received approval from the NYU Langone Health Institutional Review Board (IRB #i25-00450; 21 August 2025). All study participants signed electronic informed consent before accessing the survey. All study activities were conducted in accordance with ethical standards for research involving human participants and complied with applicable institutional and federal regulations

### 2.3. Instrument Development

#### 2.3.1. Item Generation

Guided by the Knowledge, Attitudes, and Practices (KAP) framework [35] and existing literature [15,22,23,36] and exercise guidelines [16,17], we formulated 30 items to assess knowledge of exercise and physical activity benefits, attitudes towards physical activity, and engagement in physical activity (Table 1). Fourteen of these items that assessed knowledge were pooled into the Perceived Physical Activity Benefits Scale (PBAS), while the remaining seven and nine items were pooled into the Attitudes toward Physical Activity Scale (APAS) and the Physical Activity Practice Scale (PAPS), respectively. PBAS and APAS items were rated on a five-point Likert scale ranging from strongly disagree (1) to strongly agree (5). PAPS items were rated on a five-point frequency scale ranging from never (1) to very often (5). Hence, the PBAS, APAS, and PAPS are scored from 14 to 70, 7 to 35, and 9 to 45, respectively. Higher scores indicate higher knowledge of perceived benefits, a more positive attitude, and more engagement in physical activities.

#### 2.3.2. Content Validity

Experts evaluated each item in the PABS, APAS, and PAPS for relevance using a four-point ordinal scale—not relevant (1), somewhat relevant but needs major revision (2), relevant with minor alteration (3), and extremely relevant (4). We recorded responses 3 and 4 as 1 (relevant) and responses 1 and 2 as 0 (not relevant). For each item, the Item Content Validity Index (I-CVI) was calculated as the proportion of experts endorsing the item as relevant (i.e., total relevant score divided by the total number of experts) [34]. Inter-rater agreement for each item was estimated using a Cohen’s kappa statistic (κ), calculated as $\kappa =\;(p_{o}-0.5)⁄(1-0.5)$, with $p_{o}$ representing the proportion of relevant responses for each item [37]. We retained items with an inter-rater agreement of 0.2 or higher [37]. The scale content validity index (S-CVI) was computed in two steps. First, for each expert, we calculated the proportion of relevant items (relevance proportion) on the scale by summing their individual relevance scores and dividing by the total number of items. Next, the S-CVI was computed by averaging the relevance proportions across all experts [34].

### 2.4. Analytical Plan

#### 2.4.1. Stratification

For psychometric validation, the full sample of older adults was randomly divided into derivation (33%, n = 101) and replication (67%, n = 209) samples using simple random sampling without replacement. This decision was guided by guidelines recommending 5–10 participants per item for exploratory factor analysis (7–14 participants per item across the three scales in our study) [38,39] and a minimum of 200 participants for confirmatory factor analysis to produce stable parameter estimates and reliable model fit indices [40]. The derivation sample was used for exploratory factor analysis, while the replication sample was reserved for confirmatory factor analysis.

#### 2.4.2. Missing Data

We did not encounter any missing data from the survey among the instrument experts. However, 369 older adults initiated the survey, and 310 (84%) completed it. Those who did not complete the survey completed less than 50% of the items. We performed listwise deletion for these 59 respondents because missingness was not at random [41].

#### 2.4.3. Sociodemographic Characteristics

We reported the sociodemographic characteristics of the study participants using frequency distributions and summary statistics.

#### 2.4.4. Item Analysis and Reliability

We computed the floor and ceiling effects, item difficulty, variability, discrimination, and internal consistency. Floor and ceiling effects were computed as the proportions of respondents selecting the lowest and highest response categories, respectively, for each item. Proportions below 20% are considered ideal [42]. Item difficulty and variability were assessed using the mean and standard deviation. Mid-range values are considered ideal [43]. Item discrimination was computed using the inter-item correlation, and values of 0.2 or higher are considered adequate [44]. We computed internal consistency (a measure of reliability) by calculating Cronbach’s alpha for the scale after ensuring that deleting any item would not meaningfully improve the scale’s reliability. Cronbach’s alpha value of 0.70 to 0.79 is considered adequate, 0.80 to 0.89 as good, and 0.90 and higher as excellent [45,46,47,48].

#### 2.4.5. Exploratory Factor Analysis

Using the derivation sample, we assessed the latent factors in the PBAS, APAS, and PAPS. Factors were extracted using maximum likelihood estimation. We determined the number of possible latent factors using eigenvalues greater than 1, visual inspection of scree plots, and the cumulative proportion of variance explained (exceeding 50%) [49]. We examined the factor solutions iteratively, starting from no rotation, and progressing to orthogonal and oblique rotations. The final factor solution represents the model with minimal cross-loading and the most interpretable structure. For ease of interpretation, we suppressed factor loadings with values of 0.3 or lower, consistent with prior studies [49,50,51].

#### 2.4.6. Confirmatory Factor Analysis

Using the replication sample, we examined the reliability of the factor structures identified in the exploratory factor analysis. We assessed the model fit using multiple indices, including the Comparative Fit Index (CFI), Tucker–Lewis Index (TLI), Normed Fit Index (NFI), and Root Mean Square Error of Approximation (RMSEA). Consistent with standard practice, NFI, CFI, and TLI values of 0.90–0.94 are deemed adequate fit, while values of 0.95 or higher are deemed good fit [52,53]. RMSEA values of 0.05 or less are considered a good fit, 0.05 to 0.08 an adequate fit, and 0.08 to 0.10 a marginal fit [52,53]. We limited model modifications to the item errors, guided by the standardized expected parameter change (SEPC), the modification index (MI), and the presence of a conceptual explanation for the items whose errors we allowed to covary. Specifically, we limited the SEPC to values greater than 0.2 and MI to 5, which is above the critical value of 3.84 [54]. We compared the original and modified models using the Akaike and Bayesian information criteria, with lower values indicating a better model.

### 2.5. Statistical Analysis

Data were analyzed using IBM SPSS Statistics version 28 [55] and IBM AMOS version 27 [56]. Specifically, descriptive statistics, item analysis, and exploratory factor analysis were conducted in SPSS. We conducted the confirmatory factor analyses and generated the structural equation model figures in AMOS.

## 3. Results

### 3.1. Sociodemographic Characteristics

A total of 310 older adults completed the survey, with a mean (SD) age of 70.1 (4.3) years (Table 2). Participants were predominantly female (57.1%), non-Hispanic White (50.7%), married (69.0%), and had a bachelor’s degree or higher (48.4%). Most participants lived with others (80.0%). Demographic characteristics were well balanced between the derivation and replication samples. Additionally, 11 of 15 eligible instrument experts (mean (SD) age: 31.1 (5.6) years) completed the content validity assessment of the survey items (Table 3). The experts were predominantly male (63.6%), non-Hispanic Black (72.7%), physicians (63.6%), with a mean research experience of 7.2 (2.8) years.

### 3.2. Content Validity

The PABS is a 14-item scale. None of the items required reverse scoring. The mean item and scale content validity indices were both 0.96 (Table 4). The proportion agreement and Kappa values for all 14 items ranged from 0.6 to 1.0 and 0.8 to 1.0, respectively. All 14 items were retained. The APAS is a 7-item scale. None of the items required reverse scoring. The mean item and scale content validity indices were both 0.94. The proportion agreement and Kappa values for all seven items ranged from 0.4 to 1.0 and 0.6 to 1.0, respectively. All seven items were retained. The APAS is a 9-item scale. None of the items required reverse scoring. The mean item and scale content validity indices were both 0.95. The proportion agreement and Kappa values for all nine items ranged from 0.6 to 1.0 and 0.8 to 1.0, respectively. All nine items were retained.

### 3.3. Item Analysis

All 14 items on the PABS had floor effects below 20% but exhibited ceiling effects above 20%, ranging from 57% to 75% (Table 5). The mean score of the items ranged from 4.4 to 4.7, while the item variability ranged from 0.6 to 0.8. All 14 items correlated moderately to strongly with correlation coefficients ranging from 0.5 to 0.7. The Cronbach alpha of the scale was 0.92, and deleting any item would not improve the scale’s reliability.

Similarly, all seven items on the APAS had floor effects below 20% but exhibited ceiling effects above 20%, ranging from 47% to 72%. The mean score of the items ranged from 3.9 to 4.6, while the item variability ranged from 0.6 to 1.3. All seven items correlated moderately with correlation coefficients ranging from 0.3 to 0.6. The Cronbach alpha of the scale was 0.77, and deleting any item would not improve the scale’s reliability.

Additionally, all nine items on the PAPS had floor effects below 20% but exhibited ceiling effects above 20%, ranging from 38% to 60%. The mean score of the items ranged from 3.6 to 4.3, while the item variability ranged from 0.9 to 1.5. All nine items correlated moderately to strongly with correlation coefficients ranging from 0.4 to 0.8. The Cronbach alpha of the scale was 0.91, and deleting any item would not improve the scale’s reliability.

### 3.4. Exploratory Factor Analysis

Using the derivative data, we identified two subconstructs in the PABS, generated via oblique rotation, that yielded the most parsimonious structure: the General Health Benefit subscale and the Specific Health Benefit subscale (Table 6). Eight items loaded on the General Health Benefit subscale with factor loadings ranging from 0.49 to 1.05, while six items loaded on the Specific Health Benefit subscale with factor loadings ranging from 0.46 to 0.83. The internal consistencies (Cronbach’s alpha) of the General and Specific Health Benefit subscales were 0.89 and 0.84, respectively.

Also, we identified two subconstructs in the APAS, generated via orthogonal rotation, that yielded the most parsimonious structure: the Intrinsic Motivational Factor subscale and the Extrinsic Motivational Factor subscale. Four items loaded on the Intrinsic Motivational Factor subscale with factor loadings ranging from 0.54 to 0.90, while three items loaded on the Extrinsic Motivational Factor subscale with factor loadings ranging from 0.61 to 0.89. The internal consistencies of the Intrinsic and Extrinsic Motivational Factor subscales were 0.77 and 0.80, respectively.

Additionally, we identified two subconstructs in the PAPS, generated using orthogonal rotation, that yielded the most parsimonious structure: the General Physical Activity Behavior subscale and the General Physical Activity Behavior subscale. Five items loaded on the General Physical Activity Behavior subscale with factor loadings ranging from 0.58 to 1.01, while four items loaded on the Specific Physical Activity Behavior subscale with factor loadings ranging from 0.56 to 0.74. The internal consistencies of the General and Specific Physical Activity Behavior subscales were 0.90 and 0.80, respectively.

### 3.5. Confirmatory Factor Analysis

**Figure 1:**
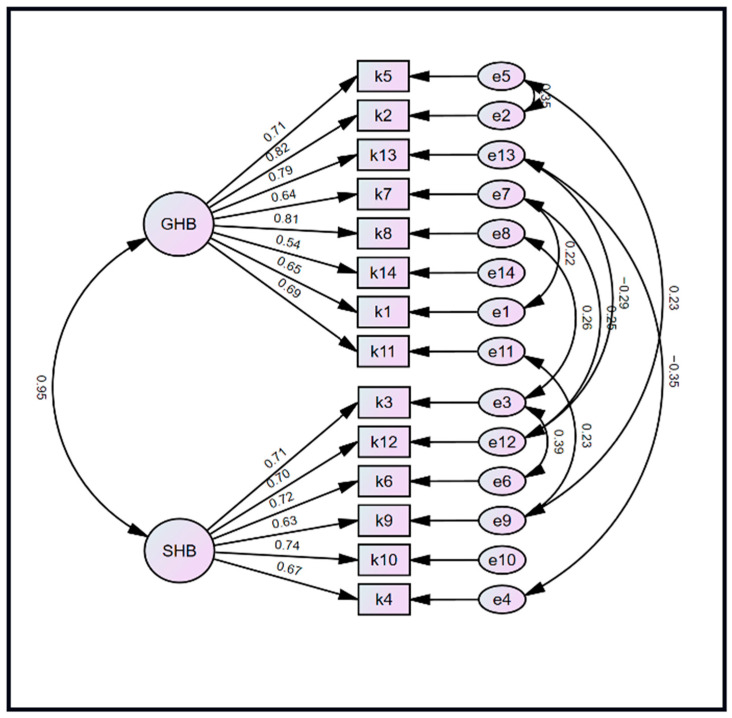
Structural equation model showing the result of the confirmatory factor analysis of the factors in the (Perceived Physical Activity Benefits for Older Adults Scale. GHB: General Health Benefit; SHB: Specific Health Benefit.

Confirmatory factor analysis of the PABS confirmed the two-subscale structure, with standardized coefficients ranging from 0.54 to 0.82 and from 0.63 to 0.74 for the General and Specific Health Benefit subscales, respectively (Figure 1). The correlation between the two factors was 0.95, and nine constraints were imposed on item errors, guided by our predefined handling of modification indices. The modified model had better fit indices compared to the baseline exploratory factor model (Table 7). The final NFI, CFI, and TLI values were borderline to good, at 0.90, 0.92, and 0.94, respectively. The RMSEA was adequate, with a value of 0.09 (90% CI: 0.07–0.10). Given the high correlation between the sub-domains of the two-factor model, we explored a unidimensional CFA model. Model comparison analyses confirmed that the two-factor model provided a superior fit to a unidimensional model at baseline and after applying an equivalent number of constraints in the modified model (modified two-factor: NFI = 0.90, CFI = 0.92, TLI = 0.94, RMSEA = 0.086, AIC = 272.98, BIC = 281.06 vs. modified one-factor: CFI = 0.90, TLI = 0.93, RMSEA = 0.093. AIC = 291.06, BIC = 298.99).

**Figure 2:**
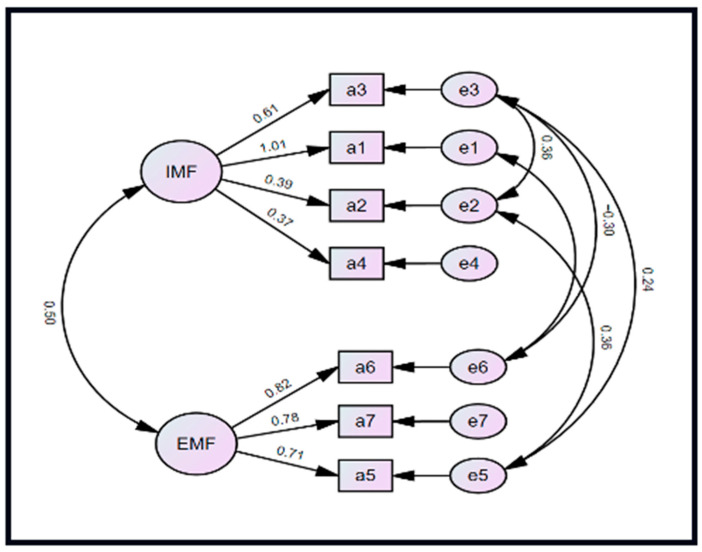
Structural equation model showing the result of the confirmatory factor analysis of the factors in the Attitude Towards Physical Activities Scale. IMF: Intrinsic Motivational Factors; EMF: Extrinsic Motivational Factors.

Also, confirmatory factor analysis of the APAS confirmed the two-subscale structure, with standardized coefficients ranging from 0.37 to 1.01 and from 0.71 to 0.82 for the Intrinsic and Extrinsic Motivational Factors subscales, respectively (Figure 2). The correlation between the two factors was 0.50, and five constraints were imposed on item errors, guided by our predefined handling of modification indices. The modified APAS model had better fit indices compared to the baseline APAS exploratory factor model. The final NFI, CFI, and TLI values were excellent at 0.97, 0.99, and 0.97, respectively. The RMSEA was adequate, with a value of 0.06 (90% CI: 0.00–0.11).

**Figure 3:**
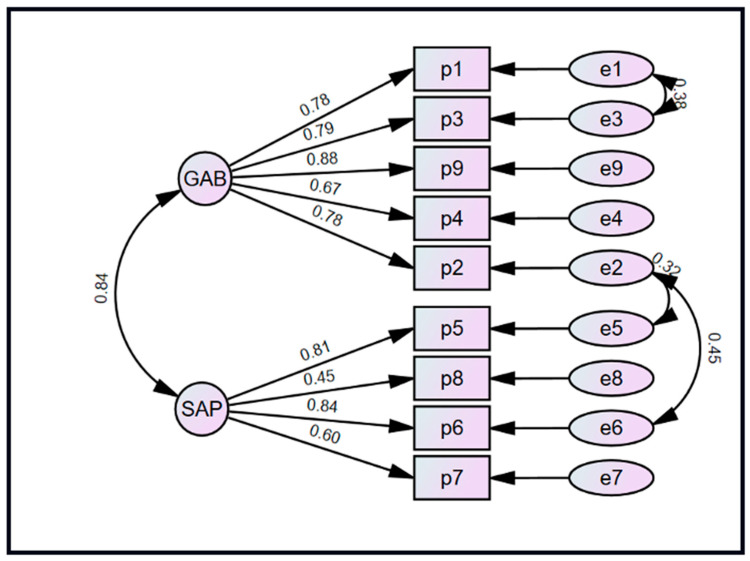
Structural equation model showing the result of the confirmatory factor analysis of the factors in the Physical Activity Practice Scale. GAB: General Activity Behavior; SAP: Structured Activity Participation.

Additionally, confirmatory factor analysis of the PAPS confirmed the two-subscale structure, with standardized coefficients ranging from 0.67 to 0.88 and from 0.45 to 0.81 for the General and Specific Physical Activity Practice subscales, respectively (Figure 3). The correlation between the two factors was 0.84, and three constraints were imposed on item errors, guided by our predefined handling of modification indices. The modified PAPS model had better fit indices compared to the baseline APAS exploratory factor model. The final NFI, CFI, and TLI values were excellent at 0.95, 0.97, and 0.95, respectively. The RMSEA was adequate, with a value of 0.08 (90% CI: 0.05–0.11).

## 4. Discussion

This study aimed to develop and validate instruments to measure perceptions, attitudes, and practices related to physical activity among older adults. Using the Knowledge, Attitudes, and Practices framework [35], we developed three complementary instruments, the PBAS, APAS, and PAPS, and evaluated their psychometric properties through a rigorous multi-step validation process. The instruments demonstrated excellent content validity, acceptable to excellent internal consistency, and stable factor structures replicated in an independent sample. Together, these findings indicate that the PBAS, APAS, and PAPS are reliable and valid tools for comprehensive assessment of perceptions, attitudes, and behaviors related to physical activity among community-dwelling older adults.

The internal consistency estimates ranged from acceptable to excellent, indicating that items within each scale and subscale coherently measured their intended constructs. Although mid-range mean score distributions are often desirable for population discrimination [43], we observed substantial ceiling effects for the PBAS, APAS, and PAPS. These high mean scores likely reflect widespread awareness of the benefits of physical activity and generally favorable attitudes toward exercise and physical activity among community-dwelling older adults, rather than poor scale performance, a pattern reported in prior studies of physical activity perception and attitudes [57,58]. Additionally, our greater score variability observed for the PAPS is consistent with extant literature demonstrating that, despite high knowledge and positive attitudes, actual engagement in physical activity remains suboptimal among older adults [9,59,60]. Given the high ceiling effects observed in the PBAS, APAS, and PAPS, these instruments may have limited ability to detect improvements in perceptions, attitudes, and practices following interventions in populations with already high baseline scores. On the contrary, these high ceiling effects make these instruments particularly useful for identifying older adults with lower perceived benefits of physical activity, poor attitudes, and reduced engagement in physical activity, who may benefit most from targeted or tailored intervention approaches.

Exploratory factor analysis identified two theoretically meaningful subscales within each measure, underscoring the multidimensional nature of older adults’ perceptions, attitudes, and practices toward physical activity. Within the PBAS, factors differentiated between General Health Benefits and Specific Health Benefits, suggesting that older adults distinguish broad, socially reinforced beliefs about exercise from more actionable, guideline- and condition-specific knowledge. This distinction may help explain the persistent discordance between high perceived benefit and low physical activity engagement reported in earlier studies [9,57,58,59,60] by identifying which subscale predicts low physical activity engagement. Similarly, the APAS separated Intrinsic Motivational Factors from Extrinsic Motivational Factors, highlighting distinct internal and external pathways that influence attitudes toward physical activity. While external motivating factors predict initiation, intrinsic motivation sustains the maintenance of such activity, consistent with the self-determination theory [61]. Additionally, the PAPS distinguished between General Activity Behavior and Structured Activity Participation, capturing differences between routine, unstructured physical activity and intentional, planned, or monitored physical activity. This differentiation identifies domains of activity that may be more amenable to targeted intervention and system-level support.

Confirmatory factor analysis supported the two-factor structure identified through exploratory analyses for all three instruments, with model fit improving following theoretically justified modifications. The near-perfect correlation between the General Health Benefit and Specific Health Benefit factors indicates that these dimensions are highly related, reflecting that older adults who recognize general benefits of physical activity are also likely to identify the specific benefits with respect to health outcomes and guidelines. Despite this high correlation, we retained the two-factor model because each factor captures conceptually distinct domains relevant for identifying perception profiles that may inform tailored messaging or intervention focus. Consistent with principles of model modification [54], we correlated error items sparingly and only between items with clear conceptual or contextual overlap. For the PBAS, correlated errors captured overlap between general and specific health benefits (K2–K5; K5–K9; K1–K7), activity guidelines and recommendations (K7–K12; K11–K9; K13–K12; K4–K13), and specific functional outcomes (K3–K6; K3–K8). For the APAS, correlated errors captured overlap between items measuring personal motivation and commitment to exercise (A2–A3), social and structural support for sustained engagement (A2–A5; A3–A5; A3–A6), and facilitative conditions for participation (A1–A6). For the Physical Activity Practice Scale, correlations reflected overlap between general activity frequency and duration (P1–P3) and between strength training and participation in structured or guided activity (P2–P5; P2–P6). These correlations are consistent with expected method variance arising from conceptual and contextual item similarity [62,63], rather than from model misspecification.

These validated instruments have several practical applications. The PBAS, APAS, and PAPS provide standardized measures to examine determinants of physical activity and assess intervention mechanisms. The subscales allow researchers to identify specific areas—such as gaps in exercise knowledge, differences in intrinsic versus extrinsic motivation, or variations in general versus structured activity engagement—enabling more targeted strategies to support behavior change. Clinically, these surveys can help identify older adults with low engagement in physical activity, including those who understand its benefits but struggle to maintain consistent or structured participation. The observed ceiling effects further highlight the instruments’ utility for targeting individuals with lower perception, less favorable attitudes, or reduced activity levels, who may benefit most from intervention efforts.

Our study has its limitations. Selection bias is possible given our online recruitment strategy, which likely captured a relatively healthy population with basic digital literacy, higher baseline physical activity levels, and greater health awareness [64,65]. This may have contributed to the observed ceiling effects, restricted score variability, and limited the generalizability of the instruments’ psychometric properties to older adults with lower health literacy or digital access. Consequently, the external validity of these instruments should be further examined in functionally and clinically diverse older adult populations, including those with chronic conditions or functional limitations. We did not assess criterion-related validity by comparing perceptions with validated knowledge measures, attitudes with behavioral intention or motivation scales, and practices with objective physical activity monitoring or established activity questionnaires. Future studies should examine these relationships to establish that the instruments predict physical activity engagement and related health outcomes. Additionally, the cross-sectional design precludes assessing test–retest reliability, which is needed to establish the instruments’ temporal stability. Future validation studies should examine test–retest reliability over appropriate time intervals to confirm score stability when participants’ underlying characteristics and constructs remain unchanged. Responses to the survey items are self-reported, and the possibility of social desirability bias and recall bias cannot be eliminated [66,67]. The cross-sectional design precludes assessment of test–retest reliability. Despite these limitations, the study has notable strengths. The instruments are grounded in a theory-informed framework, ensuring conceptual rigor. Also, the instruments address an important gap in measuring older adults’ physical activity by capturing perceptions, attitudes, and practices. The brief, self-administered format (14, 7, and 9 items respectively; approximately five minutes total) makes the instruments feasible for implementation in routine clinical encounters, community health screenings, and research settings. Additionally, identifying interpretable subscales enables assessment of specific knowledge domains, motivational factors, and types of activity engagement, making the scales particularly useful for informing tailored interventions and evaluating behavior change efforts. The interpretable subscale structure enables identification of specific deficits in knowledge domains, motivational factors, and activity engagement patterns, allowing clinicians, health educators, and researchers to develop targeted intervention strategies matched to individual needs. Furthermore, these instruments are being deployed in the ongoing ACTIVE pilot study to assess baseline characteristics of study participants, demonstrating their practical utility for both research and clinical practice.

## 5. Conclusions

The PBAS, APAS, and PAPS are reliable and valid instruments for assessing perceptions, attitudes, and physical activity practices among community-dwelling U.S. older adults. Their multidimensional structure captures both general and specific health benefits, intrinsic and extrinsic attitudes, and general and structured activity behaviors, providing a comprehensive framework for understanding determinants of physical activity among older adults. By filling a critical gap in measurement, these tools can inform research, screening, and the development of more personalized and targeted strategies to promote healthy aging through sustained physical activity. The instruments’ brief, self-administered format and successful implementation through online platforms demonstrate their suitability for digital health applications for older adults. Future research could examine the psychometric properties of these instruments in a more diverse sample of older adults with varying levels of digital literacy, chronic conditions, and functional limitations, to ensure they perform adequately across the full spectrum of physical activity engagement and health status.

## Figures and Tables

**Table 1:** Items in the Perceived Physical Activity Benefits for Older Adults Scale, Attitude Towards Physical Activities Scale, and Physical Activity Practice Scale.

ID	Perceived Physical Activity Benefits for Older Adults Scale (PABS)
K1	Regular physical activity is essential for maintaining good health in older adults.
K2	Older adults who engage in physical activity experience improved quality of life.
K3	Exercise helps older adults maintain independence in daily activities.
K4	Engaging in regular exercise reduces the risk of premature death in older adults.
K5	The benefits of exercise outweigh the risks for most older adults.
K6	Regular physical activity improves heart health and reduces the risk of cardiovascular disease.
K7	Strength training exercises help prevent osteoporosis and maintain bone health.
K8	Exercise can reduce the risk of falls by improving strength and balance.
K9	Regular exercise enhances cognitive function and reduces the risk of dementia.
K10	Physical activity can help manage chronic conditions such as diabetes and arthritis.
K11	Older adults should engage in at least 150 min of moderate-intensity physical activity per week.
K12	Strength training exercises should be performed at least twice a week for older adults.
K13	Even light-intensity activities, such as walking, provide significant health benefits for older adults.
K14	It is never too late for older adults to start exercising and gain health benefits.
ID	Attitude Towards Physical Activities Scale (APAS)
A1	I enjoy engaging in physical activity.
A2	Exercise is an important part of a healthy lifestyle.
A3	I feel motivated to exercise regularly.
A4	I believe I can still benefit from exercise regardless of my age.
A5	Exercising with others makes it more enjoyable for me.
A6	I would be more likely to exercise if I had proper guidance.
A7	Encouragement from family or friends increases my likelihood of exercising.
ID	Physical Activity Practice Scale (PAPS)
P1	I engage in physical activity at least 3 times a week.
P2	I perform strength-training exercises at least twice a week.
P3	I engage in moderate-to-vigorous physical activity for at least 150 min per week.
P4	I incorporate light physical activities (e.g., walking, stretching) into my daily routine.
P5	I participate in group-based or social exercise programs.
P6	I do balance or flexibility exercises to prevent falls.
P7	I track my physical activity levels using a device or app.
P8	I exercise regularly based on my healthcare provider’s advice.
P9	I follow an exercise plan or routine.

**Table 2:** Sociodemographic characteristics of older adults (N = 310) stratified into derivation (n = 101, 33%) and replication samples (n = 209, 67%).

Variables	Total Population	Derivation Sample	Replication Sample	*p*-Value
Mean (SD) Age	70.07 (4.29)	70.05 (4.24)	70.09 (4.44)	0.929
Sex				
Male	133 (42.90)	90 (43.06)	43 (42.57)	0.935
Female	177 (57.10)	119 (56.94)	58 (57.43)	
Race/Ethnicity				
Non-Hispanic White	157 (50.65)	106 (50.72)	51 (50.50)	0.541
Non-Hispanic Black	98 (31.61)	66 (31.58)	32 (31.68)	
Hispanic	35 (11.29)	26 (12.44)	9 (8.91)	
Other Races	20 (6.45)	11 (5.26)	9 (8.91)	
Educational Attainment				
High School or less	53 (17.10)	34 (16.27)	19 (18.81)	0.724
Some College	107 (34.52)	75 (35.89)	32 (31.68)	
Bachelor’s or higher	150 (48.39)	100 (47.85)	50 (49.50)	
Marital Status				
Married	214 (69.03)	143 (68.42)	71 (70.30)	0.255
WDS	14 (4.52)	7 (3.35)	7 (6.93)	
Never Married	82 (26.45)	59 (28.23)	23 (22.77)	
Living Situation				
Living alone	62 (20.00)	39 (18.66)	23 (22.77)	0.396
Living with others	248 (80.00)	170 (81.34)	78 (77.23)	

WDS: Widowed/Divorced/Separated.

**Table 3:** Sociodemographic characteristics of instrument experts (N = 11).

Variables	Experts (N = 11, %)
Mean (SD) Age	31.1 (5.6)
Sex	
Male	7 (63.6)
Female	4 (36.4)
Race/Ethnicity	
Non-Hispanic White	2 (18.2)
Non-Hispanic Black	8 (72.7)
Hispanic	1 (9.1)
Education	
Masters	2 (18.2)
PhD	2 (18.2)
MD	7 (63.6)
Research Experience	
Mean (SD) years	7.2 (2.8)
Profession	
Physicians	7 (63.6)
Nurses	1 (9.1)
Health Service Researchers	3 (27.3)

SD: Standard Deviation.

**Table 4:** Content validation of the items in the Perceived Physical Activity Benefits for Older Adults Scale, Attitude Towards Physical Activities Scale, and Physical Activity Practice Scale.

Items	E1	E2	E3	E4	E5	E6	E7	E8	E9	E10	E11	No in Agreement	I-CVI	Kappa	Decision
Perceived Physical Activity Benefits for Older Adults Scale															
K1	1	1	1	1	1	1	1	1	1	1	1	11	1.00	1.00	Retain
K2	1	1	0	1	1	1	1	1	1	1	1	10	0.91	0.82	Retain
K3	1	1	1	1	1	1	1	1	1	1	1	11	1.00	1.00	Retain
K4	1	1	0	1	1	1	1	1	1	1	1	10	0.91	0.82	Retain
K5	1	1	1	1	1	1	1	1	1	1	1	11	1.00	1.00	Retain
K6	1	1	1	1	1	1	1	1	1	1	1	11	1.00	1.00	Retain
K7	1	1	0	1	1	1	1	1	1	1	1	10	0.91	0.82	Retain
K8	1	1	1	1	1	1	1	1	1	1	1	11	1.00	1.00	Retain
K9	1	1	0	1	1	1	1	1	1	1	1	10	0.91	0.82	Retain
K10	1	1	1	1	1	1	1	1	1	1	1	11	1.00	1.00	Retain
K11	1	1	0	1	1	1	1	1	1	1	1	10	0.91	0.82	Retain
K12	1	1	1	1	1	1	1	1	1	1	1	11	1.00	1.00	Retain
K13	1	1	0	1	1	1	1	1	1	1	1	10	0.91	0.82	Retain
K14	1	1	1	1	1	1	1	1	1	1	1	11	1.00	1.00	Retain
Proportion Relevant	1	1	0.57	1	1	1	1	1	1	1	1	Scale CVI: 0.96, Mean I-CVI: 0.96			
Attitude Towards Physical Activities Scale															
A1	1	1	1	1	1	1	1	1	1	1	1	11	1.00	1.00	Retain
A2	1	1	1	1	1	1	1	1	1	1	1	11	1.00	1.00	Retain
A3	1	1	0	1	1	1	1	1	1	1	1	10	0.91	0.82	Retain
A4	1	1	0	1	1	1	1	1	1	1	1	10	0.91	0.82	Retain
A5	1	1	0	1	1	1	1	1	1	1	1	10	0.91	0.82	Retain
A6	1	1	0	1	0	1	1	1	1	1	1	9	0.82	0.64	Retain
A7	1	1	1	1	1	1	1	1	1	1	1	11	1.00	1.00	Retain
Proportion Relevant	1	1	0.43	1	0.86	1	1	1	1	1	1	Scale CVI: 0.94, Mean I-CVI: 0.94			
Physical Activity Practice Scale															
P1	1	1	1	1	1	1	1	1	1	1	1	11	1.00	1.00	Retain
P2	1	1	0	1	1	1	1	1	1	1	1	10	0.91	0.82	Retain
P3	1	1	1	1	1	1	1	1	1	1	1	11	1.00	1.00	Retain
P4	1	1	0	1	1	1	1	1	1	1	1	10	0.91	0.82	Retain
P5	1	1	1	1	1	1	1	1	1	1	1	11	1.00	1.00	Retain
P6	1	1	1	1	1		1	1	1	1	1	10	0.91	0.82	Retain
P7	1	1	0	1	1	1	1	1	1	1	1	10	0.91	0.82	Retain
P8	1	1	0	1	1	1	1	1	1	1	1	10	0.91	0.82	Retain
P9	1	1	1	1	1	1	1	1	1	1	1	14	1.00	1.00	Retain
Proportion Relevant	1	1	0.56	1	1	0.89	1	1	1	1	1	Scale CVI: 0.95, Mean I-CVI: 0.95			

**Table 5:** Item analysis of the Perceived Physical Activity Benefits for Older Adults Scale, Attitude Towards Physical Activities Scale, and Physical Activity Practice Scale (n = 310).

Items	Floor (n, %)	Ceiling (n, %)	Item Difficulty (Mean)	Item Variability (Std Dev)	Item Discrimination (Correlation)	Alpha If Item Deleted	Decision
*Perceived Physical Activity Benefits for Older Adults Scale*							
K1	3 (1.0)	235 (75.8)	4.70	0.637	0.652	0.917	Retain
K2	3 (1.0)	225 (72.6)	4.65	0.684	0.735	0.914	Retain
K3	4 (1.3)	220 (71.0)	4.64	0.672	0.639	0.917	Retain
K4	6 (1.9)	206 (66.5)	4.53	0.811	0.585	0.920	Retain
K5	4 (1.3)	206 (66.5)	4.54	0.786	0.716	0.915	Retain
K6	1 (0.3)	223 (71.9)	4.66	0.605	0.672	0.917	Retain
K7	2 (0.6)	222 (71.6)	4.62	0.690	0.626	0.918	Retain
K8	4 (1.3)	219 (70.6)	4.64	0.666	0.729	0.915	Retain
K9	4 (1.3)	185 (59.7)	4.42	0.843	0.625	0.918	Retain
K10	2 (0.6)	187 (60.3)	4.50	0.718	0.642	0.917	Retain
K11	4 (1.3)	181 (58.4)	4.40	0.849	0.642	0.918	Retain
K12	3 (1.0)	177 (57.1)	4.45	0.744	0.670	0.916	Retain
K13	2 (0.6)	221 (71.3)	4.65	0.646	0.698	0.916	Retain
K14	6 (1.9)	205 (66.1)	4.56	0.768	0.544	0.921	Retain
	Score Range: 14–70; Scale Mean: 63.96; Scale SD: 7.18; Cronbach Alpha: 0.92.						
*Attitude Towards Physical Activities Scale*							
A1	3 (1.0)	183 (59.0)	4.35	0.946	0.588	0.727	Retain
A2	1 (0.3)	220 (71.0)	4.61	0.719	0.420	0.760	Retain
A3	12 (3.9)	171 (55.2)	4.15	1.178	0.552	0.732	Retain
A4	2 (0.6)	223 (71.9)	4.65	0.645	0.346	0.771	Retain
A5	10 (3.2)	148 (47.7)	4.02	1.157	0.638	0.712	Retain
A6	14 (4.5)	144 (46.5)	3.92	1.252	0.406	0.770	Retain
A7	10 (3.2)	155 (50.0)	4.12	1.103	0.564	0.730	Retain
	Score Range: 7–35; Scale Mean: 29.81; Scale SD: 4.66; Cronbach Alpha: 0.77.						
*Physical Activity Practice Scale*							
P1	3 (1.0)	186 (60.0)	4.26	1.057	0.714	0.688	Retain
P2	33 (10.6)	138 (44.5)	3.74	1.405	0.778	0.681	Retain
P3	18 (5.8)	158 (51.0)	3.95	1.290	0.764	0.712	Retain
P4	1 (0.3)	173 (55.8)	4.32	0.888	0.622	0.471	Retain
P5	56 (18.1)	119 (38.4)	3.42	1.549	0.727	0.619	Retain
P6	23 (7.4)	136 (43.9)	3.81	1.299	0.765	0.675	Retain
P7	55 (17.7)	142 (45.8)	3.64	1.547	0.625	0.434	Retain
P8	23 (7.4)	153 (49.4)	3.96	1.270	0.453	0.258	Retain
P9	23 (7.4)	159 (51.3)	3.95	1.317	0.810	0.694	Retain
	Score Range: 9–45; Scale Mean: 35.05; Scale SD: 8.93; Cronbach Alpha: 0.91.						

**Table 6:** Exploratory factor analysis, subscale constructs, and subscale reliability of the items in the Perceived Physical Activity Benefits for Older Adults Scale, Attitude Towards Physical Activities Scale, and Physical Activity Practice Scale (Derivation Sample: n = 101).

Perceived Physical Activity Benefits Scale			Attitude Towards Physical Activities Scale			Physical Activity Practice Scale		
Item ID	GHB	SHB	Item ID	IMF	EMF	Item ID	GAB	SAP
K5	1.046		A3	0.897		P1	1.011	
K2	0.851		A1	0.809		P3	0.871	
K13	0.642		A2	0.697		P9	0.711	
K7	0.582		A4	0.542		P4	0.627	
K8	0.578		A6		0.894	P2	0.580	
K14	0.562		A7		0.704	P5		0.742
K1	0.547		A5		0.606	P8		0.703
K11	0.485					P6		0.685
K3		0.831				P7		0.560
K12		0.749						
K6		0.587						
K9		0.580						
K10		0.506						
K4		0.457						
Subscale Metrics *								
Measure	GHB	SHB	Measure	IMF	EMF	Measure	GAB	SAP
Mean	36.75	27.21	Mean	17.75	12.06	Mean	20.22	14.83
SD	4.30	3.30	SD	2.76	2.97	SD	5.10	4.51
Range	8–40	6–30	Range	4–20	3–15	Range	5–25	4–20
A	0.89	0.84	α	0.77	0.80	α	0.90	0.80

GHB: General Health Benefit; SHB: Specific Health Benefit; IMF: Intrinsic Motivational Factor; EMF: Extrinsic Motivational Factor; GAB: General Physical Activity Behavior; SAP: Structured Physical Activity Participation. α: Cronbach’s alpha. * Subscale metrics computed on the full sample size (n = 310).

**Table 7:** Summary of the confirmatory factor analysis of the Perceived Physical Activity Benefits for Older Adults Scale, Attitude Towards Physical Activities Scale, and Physical Activity Practice Scale (Replication Sample: n = 209).

Fit Indices	Baseline Exploratory Factor Analysis Model	Modified Confirmatory Factor Analysis Model
Perceived Physical Activity Benefits Scale		
Normed Fit Index	0.83	0.90
Comparative Fit Index	0.86	0.92
Tucker–Lewis Fit Index	0.83	0.94
RMSEA (90% CI)	0.112 (0.108–0.136)	0.086 (0.070–0.102)
AIC	395.49	272.98
BIC	402.18	281.06
χ^2^(df)	309.49 (76)	168.98 (67)
PCMIN/df	4.07	2.52
Attitude Towards Physical Activities Scale		
Normed Fit Index	0.85	0.97
Comparative Fit Index	0.87	0.99
Tucker–Lewis Fit Index	0.79	0.97
RMSEA (90% CI)	0.153 (0.121–0.187)	0.061 (0.000–0.112)
AIC	120.42	68.19
BIC	122.18	70.35
χ^2^(df)	76.42 (13)	14.19 (8)
PCMIN/df	5.88	1.77
Physical Activity Practice Scale		
Normed Fit Index	0.90	0.95
Comparative Fit Index	0.92	0.97
Tucker–Lewis Fit Index	0.89	0.95
RMSEA (90% CI)	0.125 (0.101–0.149)	0.081 (0.053–0.109)
AIC	165.95	116.39
BIC	168.78	119.52
χ^2^(df)	109.95 (26)	54.39 (23)
PCMIN/df	4.23	2.37

RMSEA: Root Mean Square Error of Approximation; AIC: Akaike Information Criteria; BIC: Bayesian Information Criteria; χ^2^(df): Chi-square (degree of freedom); PCMIN/df: Ratio of chi-square to its degrees of freedom.

## Data Availability

The original data presented in the study are openly available at FigShare: 10.6084/m9.figshare.30953792.

## Abbreviations

The following abbreviations are used in this manuscript:

PBAS	Physical Activity Benefits Scale
APAS	Attitudes toward Physical Activity Scale
PAPS	Physical Activity Practice Scale
KAP	Knowledge, Attitudes, and Practices
U.S.	United States
I-CVI	Item Content Validity Index
S-CVI	Scale Content Validity Index
CFI	Comparative Fit Index
TLI	Tucker–Lewis Index
NFI	Normed Fit Index
RMSEA	Root Mean Square Error of Approximation
GHB	General Health Benefit
SHB	Specific Health Benefit
IMF	Intrinsic Motivational Factor
EMF	Extrinsic Motivational Factor
GAB	General Physical Activity Behavior
SAP	Structured Physical Activity Participation
α	Cronbach’s alpha
κ	Cohen’s kappa statistic
